# *Where* to enhance rural palliative care? Developing a spatial model to identify suitable communities most in need of service enhancement

**DOI:** 10.1186/s12913-020-5024-y

**Published:** 2020-03-04

**Authors:** Nadine Schuurman, Michael E. Martin, Valorie A. Crooks, Ellen Randall

**Affiliations:** 1 Geography Department, Simon Fraser University, Robert C. Brown Hall, 8888 University Road, Burnaby, BC V5A 1S6 Canada; 2 University of Auckland, 23 Symonds Street, Auckland, 1142 New Zealand; 3 School of Population and Public Health, University of British Columbia, 2206 East Mall, Vancouver, BC V6T 1Z3 Canada

**Keywords:** Spatial modelling, Palliative care services, Rural, Decision support

## Abstract

**Background:**

In Canada, access to palliative care is a growing concern, particularly in rural communities. These communities have constrained health care services and accessing local palliative care can be challenging. The Site Suitability Model (SSM) was developed to identify rural “candidate” communities with need for palliative care services and existing health service capacity that could be enhanced to support a secondary palliative care hub. The purpose of this study was to test the feasibility of implementing the SSM in Ontario by generating a ranked summary of rural “candidate” communities as potential secondary palliative care hubs.

**Methods:**

Using Census data combined with community-level data, the SSM was applied to assess the suitability of 12 communities as rural secondary palliative care hubs. Scores from 0 to 1 were generated for four equally-weighted components: (1) *population* as the total population living within a 1-h drive of a candidate community; (2) *isolation* as travel time from that community to the nearest community with palliative care services; (3) *vulnerability* as community need based on a palliative care index score; and (4) *community* readiness as five dimensions of fit between a candidate community and a secondary palliative care hub. Component scores were summed for the SSM score and adjusted to range from 0 to 1.

**Results:**

Population scores for the 12 communities ranged widely (0.19–1.00), as did isolation scores (0.16–0.94). Vulnerability scores ranged more narrowly (0.27–0.35), while community readiness scores ranged from 0.4–1.0. These component scores revealed information about each community’s particular strengths and weaknesses. Final SSM scores ranged from a low of 0.33 to a high of 0.76.

**Conclusions:**

The SSM was readily implemented in Ontario. Final scores generated a ranked list based on the relative suitability of candidate communities to become secondary palliative care hubs. This list provides information for policy makers to make allocation decisions regarding rural palliative services. The calculation of each community’s scores also generates information for local policy makers about how best to provide these services within their communities. The multi-factorial structure of the model enables decision makers to adapt the relative weights of its components.

## Background

Palliative care encompasses a range of services provided to support people “living with, or dying from, advanced illness” [1] in ways that optimize the quality of their life and, ultimately, their death [1, 2]. Palliative care also extends beyond the patient, providing support to family members during end of life and bereavement; these family members, in turn, are often themselves key informal providers of palliative care [3]. Beyond family members, formal palliative services are delivered by a wide variety of providers including primary care physicians, specialist physicians, hospital staff, home care nurses, social workers, and spiritual advisors—with a goal of facilitating an end-of-life experience that is comfortable and dignified [3, 4].

Access to palliative care is a growing concern for Canada’s aging population, where the number of Canadians over 65 years of age is projected to grow to almost one in four (23%) by 2031 [5]. This growing population will necessitate adjustments in healthcare provision to ensure the range of and capacity for end-of-life services that are required to meet the needs of individuals during this life stage [6–9]. Canada, however, has lagged behind in preparing for its aging population and there are concerns about the gap between palliative care needs and available services [6, 10–12]. While the increasing demand for palliative care is now beginning to receive greater attention in research literature, there remains an insufficient focus on the particular barriers to accessing this type of care in rural communities [13, 14].

Place of residence is recognized as a social determinant of health [15, 16]. The mechanisms underwriting differences in health status between urban and rural residents are various (e.g., income, employment, socio-economic status) and include access to health care services [15]. Globally, access to palliative care within one’s home region is relatively limited in rural areas [17], and this holds true in Canada [18–20], where roughly 19% of the population lives in areas designated as rural [21].

Across Canada, rural regions are characterized by geographic isolation, vast distances between communities, and relatively small populations. These factors contribute to challenges in the recruitment and retention of health care providers; in 2016, while 19% of Canadians lived in rural communities, only 8% of physicians practiced in these communities [22]. For specialized palliative care, which ideally draws on a multi-disciplinary team of practitioners and specialists, the reality is even more stark: only 2.3% of Canada’s specialists work in rural communities [22]. Palliative care in rural settings, therefore, is seldom provided by dedicated specialists [23]. Instead, rural patients rely on generalist providers and the limited formal and informal services available in their communities [23, 24]. As palliative care increasingly moves beyond hospitals to be delivered at home, there is a need for strong community nursing capacity which is often not adequate in rural communities, and these nurses can be hindered by lack of sufficient support services and equipment [25]. These rural realities are troubling given the desire of many Canadians to die “in-place.” Further, lack of adequate community-based care can impede the wishes of rural residents who want to die at home; in Western Canada, for example, rural residents have been found to spend more time in hospital in the last year of life compared to those living in urban centres [18].

### Spatial locational analysis modelling in support of improved access to rural palliative care

Given the challenges with rural provision of palliative care services, there is a “need for innovative models of service provision” ([14], p., 256) in order to provide ready, local access to palliative care services in rural communities. In response, our research team has undertaken a multi-year research program that has resulted in the development of a spatial locational analysis model that supports the creation of secondary palliative care hubs (SPCHs) at regional levels [26–30]. Understanding the locational implications of health services as a dimension of access to adequate health care is recognized as a valuable input to decision making about siting health services [31–34], including palliative care [26]. An SPCH is a site that can enable access to local palliative care by combining existing health care provision with support from palliative care specialists in primary urban centres, through telemedicine and video-conferencing. The Site Suitability Model (SSM), which we introduce in this paper, enables the identification of rural communities with need for and community interest in palliative care services, as well as existing health service and personnel capacity, that have the potential to be enhanced to enable the creation of an SPCH [27, 28].

Working with a spatial sensibility, the SSM provides a locational analysis of rural communities with regard to their suitability as potential sites for an SPCH.

The purpose of this study was to test the feasibility of implementing the newly refined SSM in order to identify and rank a set of rural communities in the province of Ontario based on their suitability as SPCH candidates. A secondary goal was to use the ranked suitability list as a form of evidence to allocate secondary palliative care hubs.

## Methods

The assessment of a community’s suitability as a candidate site for a rural SPCH rests a community’s final SSM score. As a summative model, the SSM generates a total community score from four equally-weighted component scores.

### Components of the site suitability model

#### Population

Using readily available Canadian Census data, this component assesses need for local services and is operationalized as the total population living within a 1-h drive of a potential hub community. The working assumption is that the larger the catchment population, the larger the potential need for services. The choice of 1 h reflects considerations of barriers to patients’ and providers’ ability to reasonably travel during the end-of-life phase [27]. Travel-time catchments were generated with ArcGIS [35] using road data from the CanMap Network dataset [36] and Statistics Canada Census Blocks within each 1-h catchment aggregated to provide population counts. The population component is scored as a range from 0 to 1, based on the populations of the dissemination blocks within an hour drive time. The largest population is given a score on 1, and the remaining population centres are scored as a proportion relative to that centre.

#### Isolation

Using the ArcGIS “near facility” tool, travel times were estimated between SPCH candidate communities and the nearest community with palliative care services. This determinant of need assumes that the greater the travel time, the greater the need for access to local palliative care [27]. The isolation score is scored as a range from 0 to 1. Communities that are a minimum of 4 h driving travel time from a community with existing specialized palliative care services are given a score of 1. Communities whose travel time is less than 4 h are given proportional scores relative to the communities that scored 1.

#### Vulnerability

The vulnerability component is a community’s palliative care index (PCIX) score [30]. The PCIX incorporates four key variables associated with increased potential need for palliative care services, all drawn or generated from Statistics Canada data (Table 1).

**Table 1:** The elements of the PCIX, which is used as the vulnerability component for the SSM. See referenced papers for more detail on scoring [30, 37, 38]

Variable	Measure	Data sources
Age	Percentage of catchment population over age 75	Statistics Canada Census data
Sex	Percentage of catchment population that is female	Statistics Canada Census data
Living arrangement	Percentage of catchment population that lives alone	Statistics Canada Census data
VANDIX score	Composite of 7 dissemination area-level indicators: percentage of residents who have not completed high school; unemployment rate over age 15 years; percentage of lone-parent families; average total income; percentage of homeowners; and employment ratio [37, 38]	Statistics Canada Census data

Composite PCIX scores have a value between 0 and 1: all four variables are evenly weighted and scored between 0 and 1, then summed and divided by 4. Each PCIX element is scaled relative to the minimum and maximum values in each province; for example, the community (or communities) with the highest percentage of people over 75 is assigned a 1, and all other communities are scored relative to that community. More information on the PCIX scoring methodology is available in a paper by Schuurman et al. [30].

#### Community readiness

Community readiness is a measure of fit between an SPCH and a candidate community and introduces assessments of capacity. Operationalized as five binary Yes/No variables, it captures both community willingness and structural capacity (Table 2) [29]. Data for this component came from the Census as well as from information gathered through the review of relevant websites and by phone calls to relevant organizations.

**Table 2:** The elements of the community readiness component for the SPCH SSM. See referenced papers for more detail on scoring

Variable	Measure (Yes/No)	Data sources
Community awareness	Presence of local hospice society	Community websites; BC Hospice Palliative Care Association listings
Training and education	Presence of local college or university	Maximum 1 h drive time to college or university. List of facilities collated using the Association of Accredited Universities and Colleges of Canada website
Telemedicine utilization	Regular use of telemedicine^a^	Confirmation from administrators at local hospitals of regular telemedicine use.
Adequate supply of family doctors	Family physician to population ratio: at most 1307 persons per physician	Number of family physicians: MDSelect database Population: Statistics Canada Census data
Momentum	Expression of interest for local hospice	Confirmation from local hospice(s) of discussions or proposals for local hospice residence

^a^For the purposes of this study, telemedicine is considered the provision of clinical support to local health care providers through use of information and communication technologies [39, 40]

As a summative score, in order to achieve an overall maximum score of 1, each community readiness measure was assigned a value of 0.2 for a Yes.

### Site suitability model scoring

The SSM generates community scores by totalling the four component scores—population, isolation, vulnerability, and community readiness. Each component score was equally weighted and summed yielding an initial model score ranging from 0 to 4. Final values were scaled to 0–1. A cut-point of 0.6 is suggested as a lower bound for eligibility, based on past experience; this value is discretionary and will need testing and possible adjustment in subsequent applications of the model.

### Community selection criteria

For consideration in this study, the community selection process worked with the set of all population centres in Ontario, based on the 2011 Canadian Census [41]. We elected to implement the SSM at the provincial level because the provinces in Canada bear the primary responsibility for the provision of health care services. Ontario was selected because it is Canada’s most populous province. The first step was to reduce this set to communities with over 5000 residents [42]. This constraint ensured that a new SPCH would service a sufficient catchment population. The set of population centres was then further reduced to only include those over a 1-h drive time to the nearest palliative care centre. The definition for a palliative care centre included general hospitals with more than 500 beds, identified from the Guide to Canadian Healthcare Facilities [43], as well as dedicated hospice facilities with more than three hospice beds, identified from government and community websites and verified over the telephone. This ensured we were identifying communities without easy spatial access to palliative care services.

## Results

### Community selection

**Fig. 1:**
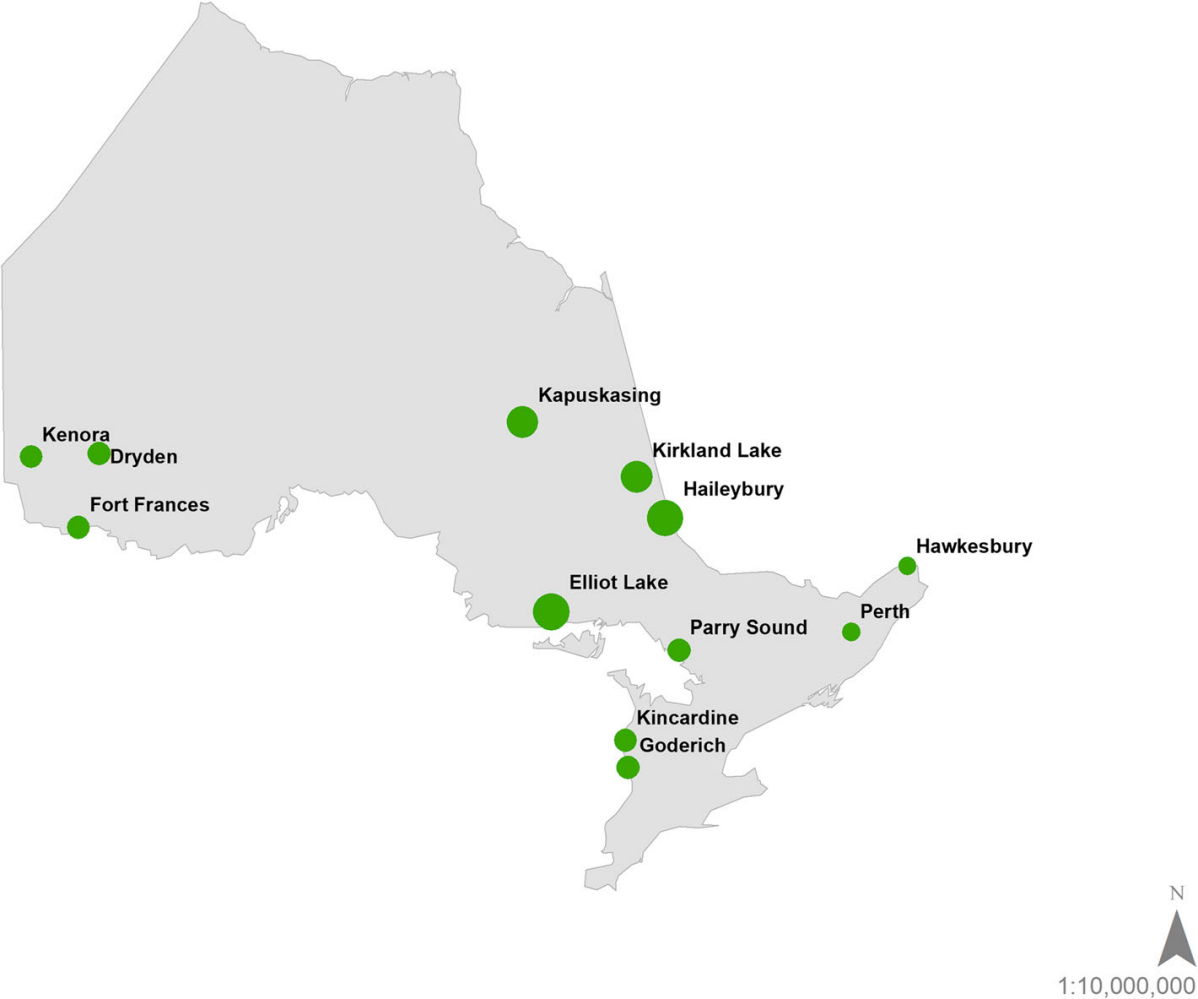
Potential SPCH sites in Ontario. The 12 communities identified as potential SPCH sites in the province of Ontario. Dot size correlates to SPCH score. This map was created by the authors in ESRI ArcGIS

The 2011 Canadian Census lists 270 population centres in Ontario; applying the criterion of a population of at least 5000 people reduced the set of possible population centres to 104 [44]. The set was further reduced by applying a 1-h drive time from the centroid of each population centre to the closest palliative care centre; using the criterion of a drive greater than 1 h to such a centre produced a final list of 12 population centres across the province of Ontario (Fig. 1). These communities, for the most part, were found in the southern regions of the province.

### Component scores

The population component scores for the 12 communities, indicating the size of local population an SPCH would serve, ranged widely from a low of 0.19 for the town of Perth to a high of 1.00 for Fort Frances. The isolation scores for these communities, signalling their relative distance from specialized palliative care services that were not available in their home communities, also ranged widely from a low of 0.16 for Hawkesbury to a high of 0.94 for Dryden.

Using the PCIX formula [30], overall vulnerability scores were generated from four sub-scores for these 12 Ontario communities. The vulnerability scores and ranged narrowly, from 0.27 to 0.35 (Table 3).

**Table 3:** Sub-scores and final vulnerability scores, based on the PCIX methodology, for potential SPCH host communities in Ontario

Vulnerability (PCIX) Scores for possible Ontario SPCH sites					
Community Name	Age (%)	Sex (%)	Living Arrangement (%)	VANDIX	VulnerabilityScore
**Dryden**	0.08	0.46	0.08	0.53	0.29
**Elliot Lake**	0.13	0.46	0.12	0.67	0.35
**Fort Frances**	0.10	0.46	0.10	0.52	0.30
**Goderich**	0.11	0.49	0.09	0.47	0.29
**Haileybury**	0.12	0.51	0.10	0.67	0.34
**Hawkesbury**	0.09	0.50	0.08	0.48	0.28
**Kapuskasing**	0.10	0.47	0.12	0.64	0.33
**Kenora**	0.09	0.42	0.09	0.55	0.29
**Kincardine**	0.12	0.50	0.10	0.49	0.30
**Kirkland Lake**	0.10	0.46	0.10	0.63	0.32
**Parry Sound**	0.18	0.47	0.10	0.52	0.30
**Perth**	0.08	0.43	0.08	0.49	0.27

The community readiness scores for the Ontario communities are presented in Table 4. The municipality of Parry Sound is the only population centre to achieve the maximum score of 1. The sub-scores reveal that community awareness is largely present across the set of communities, as are training and education resources. Telemedicine is being used in all communities and nearly all of the population centres had adequate access to physicians, with the exception of Haileybury. The momentum scores, however, show that formal initiatives towards local palliative services are largely lacking.

**Table 4:** Community Readiness scores for potential SPCH host communities in Ontario

Community Readiness Scores for possible Ontario SPCH sites						
Community Name	Community awareness	Training/education	Telemedicine	Adequate supply of FPs	Momentum	Score
**Dryden**	0.2	0.2	0.2	0.2	0	0.8
**Elliot Lake**	0.2	0	0.2	0.2	0	0.6
**Fort Frances**	0.2	0.2	0.2	0.2	0	0.8
**Goderich**	0.2	0	0.2	0.2	0	0.6
**Haileybury**	0.2	0.2	0.2	0	0	0.6
**Hawkesbury**	0	0.2	0.2	0.2	0	0.6
**Kapuskasing**	0	0	0.2	0.2	0	0.4
**Kenora**	0.2	0.2	0.2	0.2	0	0.8
**Kincardine**	0.2	0.2	0.2	0.2	0	0.8
**Kirkland Lake**	0.2	0.2	0.2	0.2	0	0.8
**Parry Sound**	0.2	0.2	0.2	0.2	0.2	1.0
**Perth**	0.2	0.2	0.2	0.2	0	0.8

### Site suitability model scores

Scores for the 12 communities (Table 5) ranged from a low of 0.33 for Hawkesebury to a high of 0.76 for Fort Frances, indicating the latter may be the most suitable location of this particular set of communities for further investigation and investment. Three communities had scores above the preliminary cut-point of 0.6, designated as the proposed threshold for suitability. Having the component scores reveals where communities have weaknesses and strengths, and these can vary across site with the same score. For example, Kapuskasing and Kincardine have identical SSM scores, but their component scores reveal that while Kincardine had a fairly high community readiness score, Kapuskasing did not.

**Table 5:** Summary of the SSM component scores and final SPCH SSM scores. Component scores are summed and divided by four to generate the final scores

SSM Scores for possible Ontario SPCH sites					
Community Name	Population Score	Isolation Score	VulnerabilityScore	Community Readiness Score	SSM Score
**Dryden**	0.81	0.94	0.29	0.80	0.71
**Elliot Lake**	0.68	0.45	0.35	0.60	0.52
**Fort Frances**	1.00	0.93	0.30	0.80	0.76
**Goderich**	0.23	0.22	0.29	0.60	0.34
**Haileybury**	0.56	0.41	0.34	0.60	0.48
**Hawkesbury**	0.26	0.16	0.28	0.60	0.33
**Kapuskasing**	0.46	0.44	0.33	0.40	0.41
**Kenora**	0.87	0.56	0.29	0.80	0.63
**Kincardine**	0.25	0.27	0.30	0.80	0.41
**Kirkland Lake**	0.26	0.26	0.32	0.80	0.41
**Parry Sound**	0.21	0.25	0.30	1.00	0.44
**Perth**	0.19	0.22	0.27	0.80	0.37

## Discussion

The SSM represents a data-driven approach to identifying potential sites for SPCHs in rural areas that incorporates multiple factors that affect a community’s need for and appropriateness as a potential SPCH hub. Developed and tested in the province of BC, the latest model was tested in the province of Ontario, demonstrating its portability and utility in any context where there are readily accessible Census, spatial, and local resource data to generate the component and final SSM scores. The model, therefore, enhances the ability of provincial health care decision makers to utilize these types of publicly available data to generate assessments of rural communities’ suitability as potential SPCH sites which can provide much needed local end-of-life resources for aging ex-urban populations in Canada and beyond. Though designed and tested in individual provinces in Canada, this model has transferability to other contexts that face similar challenges in rural health service provision, such as Australia and the United States.

The results detailed for the Ontario communities reveal the architecture of the SSM. Constructed using a set of key information-rich components, the model returns a corresponding set of component sub-scores that extend the model’s value. Each component on its own provides valuable information for health care decision makers about key aspects of the populations they are trying to serve. This is particularly apparent in a component like community readiness, which provides stakeholders with key information about a community’s relative strengths and weaknesses in this domain. Kelly et al. [24], for example, identified insufficient community-level understanding of palliative care as a potential challenge to overcome in the development of local rural palliative services. The community readiness component has indicators that speak directly to this need for local understanding—e.g., the community awareness indicator—and can aid decision makers in pinpointing particular vulnerabilities within their communities that need prioritizing.

Further, the model is inherently flexible. Components can be modified, dropped, or added according to site-specific priorities. Similarly, weights can be valued and revalued based on evolving resources and population needs. For example, in the Atlantic province of Newfoundland & Labrador the factor of drive time might benefit from being adjusted or weighted differently in acknowledgement of the impact of the coastal environment on the road network [45]. This adaptability facilitates a critical input to ensuring successful implementation of the tool at the provincial or local level, and ultimately, the validation of its output—namely, the expert knowledge of local stakeholders. These decision makers can draw on their experience and expertise to recalibrate the SSM to best represent the current reality within their communities.

Given the importance of “on-the-ground” knowledge and expertise to the optimal calibration of the SSM, future research with this model is intended in order to explore the “fit” between SSM community scores and community stakeholders’ perceptions of their communities’ actual suitability and viability for becoming an SPCH. A series of case studies comprising interviews with key stakeholders is envisioned.

### Limitations and considerations

One of the key strengths of the SSM, the ability to adjust weights to reflect individual local contexts, is also a potential shortcoming in that the model does not offer a “one-size-fits-all” weighting system that is sure to be appropriate across all communities. Deployment of the tool at a provincial level across multiple communities, for example, will require input from community-level stakeholders who will need to bring their expert knowledge to bear on assigning appropriate weights to the model’s components. The SSM may also not always account for particular contextual factors that could influence the delivery of palliative care within individual rural communities, for instance a factor such as health system bureaucracy [24]. Interpreting the community scores within the context of a larger structural influences such as the health system will again depend on the expertise of the decision makers tasked with making decisions about allocating palliative care resources.

## Conclusions

The SSM is the product of over a decade of iterative development to create a nuanced and flexible model that can draw upon available public data and readily collected local data to generate information that will aid decision makers at community and provincial levels to identify a community’s suitability as an SPCH site. The model is information-rich: given its multi-factorial structure, even if communities are not ultimately selected as SPCH sites, the calculation of their population, isolation, vulnerability, and community readiness component scores provides policy makers and other stakeholders with key information that can inform decisions about resource allocation. Given the multi-factorial structure of the model, the SSM will enable decision makers to adapt the relative weights of its components to generate scores that reflect their individual knowledge about the needs and resources of their jurisdictions.

## Data Availability

1) Statistics Canada Census of the Population, and GIS Data, as facilitated through the University of Toronto CHASS Database: StatsCan Data: https://www150.statcan.gc.ca/n1/en/type/data?MM=1 CHASS: http://datacentre.chass.utoronto.ca/. This is a closed dataset, open only to Canadian University researchers. 2) DMTI CanMap Route Logistics network Datasets: https://www.dmtispatial.com/canmap/. This is a closed dataset, open only to Canadian University researchers. 3) AUCC lists of Canadian university and colleges: https://www.aucc.ca/. This is an open dataset. 4) Scott’s Directories MD Select Canadian Medical Directory: https://www.mdselect.ca/canadian-doctors-directory/. This dataset is available to purchase. 5) Community hospice societies: This data was gathered from individual community websites, direct calls to hospitals and practitioners as well as direct calls to Telehealth Ontario. https://www.ontario.ca/page/get-medical-advice-telehealth-ontario . These data are freely a.

## Abbreviations

BC: British Columbia
PCIX: Palliative Care Index
SPCH: Secondary Palliative Care Hub
SSM: Site Suitability Model
